# COVID-19–Related Insurance Coverage Changes and Disparities in Access to Care Among Low-Income US Adults in 4 Southern States

**DOI:** 10.1001/jamahealthforum.2021.2007

**Published:** 2021-08-13

**Authors:** Jose F. Figueroa, Peggah Khorrami, Aditi Bhanja, E. John Orav, Arnold M. Epstein, Benjamin D. Sommers

**Affiliations:** 1 Department of Health Policy and Management, Harvard T.H. Chan School of Public Health, Boston, Massachusetts; 2 Department of Medicine, Brigham & Women’s Hospital, Boston, Massachusetts

## Abstract

**Importance:**

It is unclear how the COVID-19 pandemic and its associated economic downturn have affected insurance coverage and disparities in access to health care among low-income families and people of color in states that have and have not expanded Medicaid.

**Objective:**

To determine changes in insurance coverage and disparities in access to health care among low-income families and people of color across 4 Southern states and by Medicaid expansion status.

**Design, Setting, and Participants:**

This random-digit dialing telephone survey study of US citizens ages 19 to 64 years with a family income less than 138% of the federal poverty line in in 4 states (Arkansas, Kentucky, Louisiana, and Texas) was conducted from October to December 2020. Using a difference-in-differences design, we estimated changes in outcomes by Medicaid expansion status overall and by race and ethnicity in 2020 (n = 1804) compared with 2018 to 2019 (n = 5710). We also explored barriers to health care and use of telehealth by race and ethnicity. Data analysis was conducted from January 2021 to March 2021.

**Exposures:**

COVID-19 pandemic and prior Medicaid expansion status.

**Main Outcomes and Measures:**

Primary outcome was the uninsured rate and secondary outcomes were financial and nonfinancial barriers to health care access.

**Results:**

Of 7514 respondents (11% response rate; 3889 White non-Latinx [51.8%], 1881 Black non-Latinx [25.0%], and 1156 Latinx individuals [15.4%]; 4161 women [55.4%]), 5815 (77.4%) were in the states with previous expansion and 1699 (22.6%) were in Texas (nonexpansion state). Respondents in the expansion states were older, more likely White, and less likely to have attended college compared with respondents in Texas. Uninsurance rate in 2020 rose by 7.4 percentage points in Texas (95% CI, 2.2-12.6; *P* = .01) and 2.5 percentage points in expansion states (95% CI, −1.9 to 7.0; *P* = .27), with a difference-in-differences estimate for Medicaid expansion of −4.9% (95% CI, −11.3 to 1.6; *P* = .14). Among Black and Latinx individuals, Medicaid expansion was associated with protection against a rise in the uninsured rate (difference-in-differences, −9.5%; 95% CI, −19.0 to −0.1; *P* = .048). Measures of access, including having a personal physician and regular care for chronic conditions, worsened significantly in 2020 in all 4 states, with no significant difference by Medicaid expansion status.

**Conclusions and Relevance:**

In this survey of US adults, uninsured rates increased among low-income adults in 4 Southern states during the COVID-19 pandemic, but Medicaid expansion states, that association was diminished among Black and Latinx individuals. Nonfinancial barriers to care because of the pandemic were common in all states.

## Key Points

**Question:**

How did the COVID-19 pandemic and its associated economic recession affect insurance coverage, disparities in access to health care, and affordability of care among low-income families, and did this pattern vary by Medicaid expansion status?

**Findings:**

In this survey study of US adults, uninsured rates rose among low-income adults in 4 Southern states (Arkansas, Kentucky, Louisiana, and Texas) during the COVID-19 pandemic, but in states with Medicaid expansion, uninsured rate increases were more moderate among Black and Latinx individuals. Nonfinancial barriers to care because of the pandemic were common in all states.

**Meaning:**

These findings suggest that the COVID-19 pandemic affected insurance coverage and the ability of low-income people to access health care, but it appears that the presence of Medicaid expansion was protective for Black and Latinx individuals.

## Teaser

This survey study examines changes in insurance coverage and disparities in access to health care among low-income families and people of color across 4 southern states and by Medicaid expansion status before and during the COVID-19 pandemic.

## Introduction

The COVID-19 pandemic and associated economic recession in 2020 led to large disruptions in employment and health care use for millions of Americans. These disruptions threaten to worsen access to care,^1^ particularly among communities of color who have experienced high infection rates and greater job losses because of various structural inequities and racism that affected housing, employment, incarceration, and nursing home quality.^2,3,4^ While a recent survey indicated that erosions of coverage during early 2020 may not have been as large as initially feared,^1^ the precise association of these dual health and economic crises with overall rates of health insurance coverage and access to care remains unclear given the lack of a validated real-time tracking system for the uninsured population.^5^

The COVID-19 pandemic also presented the first major challenge to the US health insurance system since the implementation of the US Affordable Care Act (ACA). Before the pandemic, ACA appeared to be significantly associated with a reduced risk of people becoming uninsured after job loss^6^; more broadly, the law led to improved coverage, access to care, and health in multiple domains.^7,8,9,10^ However, it is unclear how these results will extrapolate to 2020 given the unprecedented job losses across the US. To date, to our knowledge, there is little published evidence that examines whether Medicaid expansion has better protected coverage for low-income people or helped prevent a worsening of racial and ethnic disparities during the pandemic. Additionally, it is not clear whether low-income people of color have disproportionately experienced increased barriers to health care during the pandemic, including access to clinicians, use of telehealth services, and affordability of care. Before the pandemic, Black and Latinx people historically reported more of these financial and nonfinancial barriers to care,^11^ raising concern that the pandemic may have potentially worsened these disparities.

In this study, we sought to examine 3 questions. First, how was the COVID-19 pandemic associated with insurance coverage, access to care, and affordability of care among low-income adults, and how did this pattern vary by Medicaid expansion status? Second, how do these patterns differ in analyses stratified by race and ethnicity? Finally, did self-reported barriers to health care use, including access to telehealth, differ significantly by race and ethnicity during the pandemic?

## Methods

### Study Design, Setting, and Sample

Using a nonpartisan survey vendor (Social Science Research Solutions), we conducted a random-digit dialing telephone survey of low-income adults in 4 Southern states (Arkansas, Kentucky, Louisiana, and Texas) between October 5, 2020, and December 23, 2020. The study was approved by the Harvard T.H. Chan School of Public Health institutional review board. Participation in this survey was voluntary, and verbal informed consent was provided by respondents during the telephone interview. This survey study followed the American Association for Public Opinion Research (AAPOR) reporting guideline. The first 3 states expanded Medicaid under ACA (Arkansas and Kentucky in 2014, Louisiana in 2016), whereas Texas has not expanded Medicaid. Because prior work suggested that ACA coverage gains had largely plateaued by 2018, we used baseline data from prior waves of the survey that were collected between November and December of 2018 and 2019 (the pre–COVID-19 period) as a baseline for evaluating changes during COVID-19 in 2020 (the postperiod) and the potential differences in these changes in states with prior Medicaid expansion. A difference-in-difference analysis was used to compare changes over time in the 3 states that had previously expanded Medicaid vs Texas (the nonexpansion state).

The sample included landline and mobile phone users, and the survey was available in English and Spanish. Inclusion criteria were US citizens ages 19 to 64 years who reported a family income in the prior year of less than 138% of the federal poverty level. Inclusion was based on income during the previous year (2019) to prevent any major changes in the composition of the sample (compared with the 2018-2019 baseline data) because it is possible that the pandemic-related job losses in 2020 were associated with shifts in the types of people who were considered low income in 2020. We oversampled in Texas and Louisiana to ensure adequate representation of Black and Latinx respondents, but all analyses were weighted to state benchmarks, as described later in the article, to produce population-representative estimates.

All interviews were conducted using Social Science Research Solutions’ computer-assisted telephone interviewing system, which ensures that questions follow logical skip patterns. To maximize survey responses, each nonresponsive telephone number that was not already set up with a callback (ie, answering machines, no answers, and/or busy) was contacted up to 4 times, varying the times of day and days of the week. All numbers with initial refusals were recontacted by interviewers who specialize in refusal conversation. Voicemail messages were left for those with a callback option with information about the survey. For additional details on survey approach, see eMethods 1 in the Supplement.

The survey instrument collected data on health insurance coverage, access to health care, health status, employment, and demographic information. Information on race and ethnicity was categorized in similar categories as the American Community Survey to allow for appropriate population weighting in our analyses. Respondents were asked if they considered themselves of Latinx or Hispanic origin or descent, such as Mexican, Puerto Rican, Cuban, or some other Latin American background. Information on race was obtained by asking respondents whether they considered themselves as a White, Black or African American, Asian, American Indian or Alaska Native, Native Hawaiian or other Pacific Islander, or other category individual. Questions for outcomes that were tracked over time were unchanged from previous versions of this survey, which was validated using federal government survey data, which showed that our approach produced similar state estimates and trends for coverage and other key study outcomes.^12^ New survey items that focused on the COVID-19 pandemic were adapted from the US Census Bureau’s Pulse survey and other published research.^5,13^

### Primary and Secondary Outcomes

The primary outcome was the number of adults without any health insurance coverage, which has been collected longitudinally in prior surveys. Secondary outcomes included the type of health insurance coverage, measures of access (eg, having a personal physician, having a usual source of care [not including the emergency department]), having regular care for chronic conditions (limited to those reporting any of 7 chronic conditions: hypertension, coronary artery disease, asthma/chronic obstructive pulmonary disease [COPD], diabetes, depression or anxiety, cancer, or substance use disorder), and measures of affordability (eg, delaying needed care because of cost, skipping medications because of cost, difficulty with medical bills). The specific wording of each survey question is provided in eMethods 2 in the Supplement.

We also asked a series of questions about whether respondents had waited to seek medical care or chosen not to seek medical care during the past year for any of the following reasons (respondents could choose more than 1): (1) difficulty affording care; (2) fear of contracting COVID-19; (3) the physician’s office was closed; (4) lack of access to telemedicine; (5) no access to transportation; and (6) too busy with work, childcare, or caring for a family member.

### Statistical Analyses

We used a difference-in-difference model to assess whether prior Medicaid expansion mitigated the effect of the pandemic and recession in 2020 compared with 2018 to 2019. The coefficient of interest was the interaction between Medicaid expansion status and the year 2020. The model used state fixed effects and a dummy for the year 2020 and further controlled for age, sex, race and ethnicity, education, marital status, and urban vs rural residence. Robust standard errors were clustered at the county level, following previous research using this data set.^12,14^ The analysis used a linear regression model for ease of interpretation of the key interaction term.^15^ We then repeated our main model as stratified by race and analyzed results for Black and Latinx individuals separately from White non-Latinx individuals (other racial groups comprised less than 10% of the sample, precluding a stratified model of those groups). For descriptive purposes only, we also presented long-term trends in coverage using our survey back to 2013, but we did not include years before 2018 in our regression modeling because of the large coverage and access effects associated with ACA’s initial coverage expansion.^14^ For all indicators, the full sample was used except for 1 measure, regular care for chronic condition, for which the sample was limited only to patients who reported at least 1 of the following conditions: hypertension, heart attack/coronary artery disease, stroke, asthma/COPD, diabetes, depression or anxiety, cancer, and substance use disorder.

Next, we conducted a cross-sectional multivariable regression model that examined the prevalence of barriers to care in 2020. A logistic regression model was used for each outcome (ie, whether a particular barrier was reported), with the sample limited to those who reported delaying care during the past year, and odds ratios were then converted to predicted probabilities. We first assessed for racial and ethnic disparities for each outcome by including non-Latinx White vs Black or Latinx race as the only primary predictor. We then specified a fully adjusted model, with independent variables, including race and ethnicity, age, education, marital status, urban vs rural state, presence of any chronic conditions, state of residence, and county-level cumulative rate of COVID infections per 100 000 during 2020.^16^ A detailed description of our regression equations can be found in eMethods 3 in the Supplement.

All analyses were weighted using state-specific benchmarks that were derived from federal survey data for the demographic variables listed in the previously described regression models. See eMethods 1 in the Supplement for additional details on survey design, weighting, response rate, and regression models. As a sensitivity analysis, we performed a pretrend test that compared 2019 vs 2018 across the different outcomes by expansion status before the pandemic. Analyses used Stata, version 16.0 (StataCorp), and *P* values were considered significant at the <.05 level.

## Results

### Sample Characteristics

The sample included 7514 respondents across the 3 years of the sample, comprising 1804 in 2020, 2706 in 2019, and 3004 in 2018. The overall response rate was 11%. Table 1 presents descriptive statistics for the sample, stratified by Texas (nonexpansion state) vs Kentucky, Arkansas, and Louisiana (expansion states). The state populations differed by age, race and ethnicity, rural/urban location, interview conducted in Spanish, and education. In the Medicaid expansion states, there were fewer younger people between ages 19 to 29 years than in Texas (11.9% vs 16.0%), but more people ages 60 to 64 years (18.4% vs 12.7%).

**Table 1.** Descriptive Statistics of the Study Sample

Variable	AR, KY, and LA (Medicaid expansion states)	TX (nonexpansion state)
Sample size, No.^a^	5815	1699
Age, y		
19-29	693 (11.9)	271 (16.0)
30-39	961 (16.5)	278 (16.4)
40-49	1280 (22.0)	397 (23.4)
50-59	1737 (29.9)	507 (29.8)
60-64	1068 (18.4)	215 (12.7)
Race and ethnicity		
Black non-Latinx	1650 (28.4)	231 (13.6)
Latinx	289 (5.0)	867 (51.0)
Other^b^	474 (8.2)	114 (6.7)
White non-Latinx	3402 (58.5)	487 (28.7)
Education		
Less than high school degree	1263 (21.7)	368 (21.7)
High school graduate	2454 (42.2)	573 (33.7)
Some college	2098 (36.1)	758 (44.6)
Other variables		
Women	3200 (55.0)	961 (56.6)
Men	2615 (45.0)	738 (43.4)
Married or living with a partner	2185 (37.6)	613 (36.0)
Spanish-language interview	31 (0.5)	204 (12.0)
Rural	3191 (54.9)	353 (20.8)

Abbreviations: AR, Arkansas; KY, Kentucky; LA, Louisiana; TX, Texas.

^a^ Study sample was derived from a telephone survey of low-income adults ages 19 to 64 years conducted in November to December 2018 and 2019 and October to December 2020. All estimates are survey-weighted as described in the Methods section.

^b^ The Other racial category included people who identified as Asian, American Indian or Alaska Native, Native Hawaiian or Pacific Islander, or simply stated “Other.”

The Figure shows the percentage of each state’s sample that lacked health insurance by year. The figure included baseline data from 2013 to 2016 from previously published analyses^14^; Louisiana was not added to this survey until 2016. The uninsured rate started at nearly 40% in 2013 in the study states, but then fell rapidly in the Medicaid expansion states starting in 2014 when the Medicaid expansion took effect. The uninsured rate fell more modestly in Texas. By 2018, coverage changes were leveling off in all 4 states, although with a slight decline still evident in Louisiana, which expanded in 2016. Between 2019 and 2020, the uninsured rate increased from 29.4% to 34.8% in Texas and from 14.2% to 16.1% in Arkansas; it was stable (9.2% vs 9.3%) in Kentucky; and it fell from 11.9% to 8.9% in Louisiana.

**Figure.**
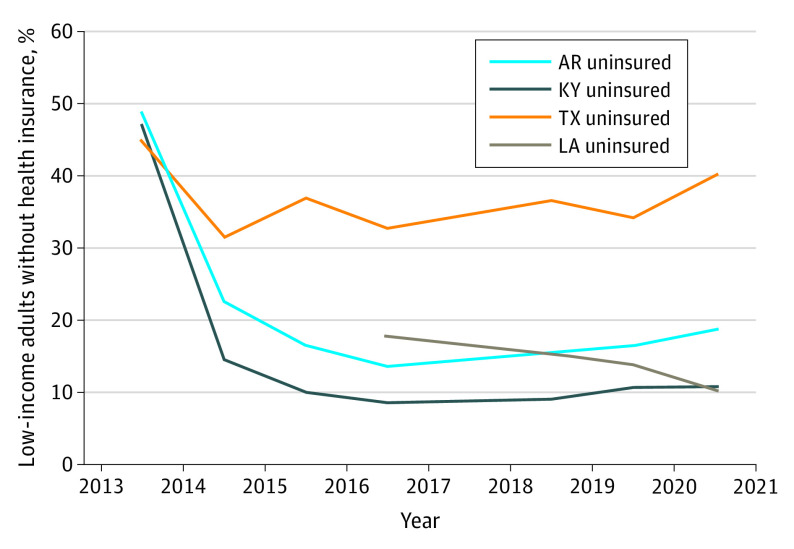
Percentage of Low-Income Adults Without Health Insurance by State From 2013 to 2020 Based on the authors’ analysis of telephone survey data from Arkansas (AR), Kentucky (KY), Louisiana (LA), and Texas (TX) from 2013 to 2020 of US citizens ages 19 to 64 years with family incomes below 138% of the federal poverty level. No survey data were collected in 2017, so this data point is a linear extrapolation of 2016 and 2018. Louisiana was added to the survey in 2016.

Table 2 presents adjusted changes in health insurance, access to care, and affordability of care associated with living in a Medicaid expansion state during the 2020 COVID-19 pandemic. In Medicaid expansion states, more respondents reported uninsurance (+2.5 percentage points), with minimal changes in coverage from Medicaid and Marketplace, whereas fewer respondents reported coverage under employer-sponsored insurance (−1.7 percentage points) and other health insurance (−3.9 percentage points) in 2020 compared with 2018 to 2019. However, these changes over time were not statistically significant. The nonexpansion state had a significant increase in uninsurance of 7.4 percentage points (*P* = .01) and a significant decrease in coverage under other health insurances (−5.1 percentage points; *P* = .01). The differences in coverage changes between states that did and did not expand Medicaid were not statistically significant.

**Table 2.** Changes in Coverage and Access to Care in 2020 vs 2018 to 2019 in Association With Medicaid Expansion^a^

Outcome	Arkansas, Kentucky, and Louisiana (Medicaid expansion states)		Texas (nonexpansion state)		Difference-in-differences estimate	
	2020 vs 2018-2019, %	*P* value	2020 vs 2018-2019, %	*P* value	Net change, % (95% CI)	*P* value
Coverage						
Uninsured	2.5	.27	7.4	.01	−4.9 (−11.4 to 1.6)	.14
Medicaid or marketplace	0.1	.97	−4.3	.09	4.4 (−2.4 to 11.2)	.22
Employer-sponsored insurance	−1.7	.44	−0.2	.90	−1.5 (−7.0 to 4.0)	.59
Other health insurance	−3.9	.07	−5.1	.01	1.2 (−4.0 to 6.3)	.66
Access to care						
Has a personal physician	−11.3	<.001	−14.0	<.001	2.7 (−5.0 to 10.4)	.49
Usual source of care	−5.1	.04	−7.0	.003	1.9 (−4.1 to 8.0)	.53
Regular care for chronic condition^b^	−10.5	<.001	−10.3	.03	−0.2 (−10.8 to 10.4)	.97
Affordability of care						
Skipped medication because of cost	−9.0	<.001	−13.1	<.001	4.1 (−2.7 to 10.9)	.23
Trouble paying medical bills	−9.4	.001	−5.4	.08	−4.0 (−11.6 to 3.5)	.30
Cost-related delay in care	−5.2	.03	2.3	.56	−7.5 (−16.4 to 1.5)	.10

^a^ Results show survey-weighted difference-in-differences estimates for expansion states (Arkansas, Kentucky, and Louisiana) vs Texas. All analyses adjusted for sex, age, race and ethnicity, marital status, education, urban vs rural residence, and state. The sample contained 7515 US citizens ages 19 to 64 years with family incomes at or below 138% of the federal poverty level (minus item nonresponse for each specific outcome), except where otherwise noted.

^b^ For the regular care for chronic condition measure, the sample was limited to patients who reported at least 1 of the following conditions: hypertension, heart attack/coronary artery disease, stroke, asthma/chronic obstructive pulmonary disease, diabetes, depression or anxiety, cancer, and substance use disorder (n = 5687).

From 2018 to 2019 to 2020, Medicaid expansion and nonexpansion states showed significant decreases in access to care. Having a personal physician decreased by 11.3 percentage points (*P* < .001) and 14.0 percentage points (*P* < .001), respectively. The changes in the expansion states were not significantly different than those in the nonexpansion states. Regarding the affordability of care, expanded states had significant decreases in the proportion of respondents who skipped medications because of cost (−9.0 percentage points; *P* < .001), had trouble paying medical bills (−9.4 percentage points; *P* < .001), and experienced cost-related delays in care (−5.2 percentage points; *P* = 0.25) in 2020. Comparatively, Texas (the nonexpansion state) also had a significant decrease in reported skipped medications (13.1 percentage points; *P* < .001) but no significant changes in trouble paying medical bills or delays in care. None of the difference-in-difference estimates of affordability between expanded and nonexpanded states were statistically significant. Baseline rates and unadjusted, weighted results can be found in eTable 1 in the Supplement.

Table 3 presents changes in coverage and access that were associated with previous Medicaid expansion separately for Black and Latinx and White individuals. Across coverage outcomes, Black and Latinx individuals reported significantly less uninsurance (−9.5 percentage points, *P* = .048) and significantly more coverage under other health insurance (6.8 percentage points; *P* = .03) in 2020 in Medicaid expansion states compared with Texas (nonexpansion state). Baseline means and adjusted differences by period and expansion status can be found in eTable 2 in the Supplement for Black and Latinx respondents and eTable 3 in the Supplement for White respondents.

**Table 3.** Changes in Coverage and Access to Care in 2020 vs 2018 to 2019 in Association with Medicaid Expansion ny Race and Ethnicity^a^

Outcome	Difference-in-differences			
	White non-Latinx (n = 3889)		Black and/or Latinx (n = 3037)	
	Net change, % (95% CI)	*P* value	Net change, % (95% CI)	*P* value
Coverage				
Uninsured	1.6 (−10.2 to 13.5)	.76	−9.5 (−19.0 to −0.10)	.048
Medicaid or marketplace	6.8 (−5.6 to 19.2)	.28	0.6 (−9.3 to 10.6)	.90
Employer-sponsored insurance	−4.0 (−14.9 to 6.9)	.47	0.8 (−7.0 to 8.6)	.84
Other health insurance	−3.1 (−12.1 to 5.9)	.50	6.8 (0.7 to 12.8)	.03
Access to care				
Has a personal physician	2.2 (−12 to 2 16.6)	.76	2.3 (−9.6 to 14.2)	.70
Usual source of care	0.3 (−10.3 to 10.8)	.96	−0.1 (−9.6 to 9.3)	.98
Regular care for chronic condition^b^	2.7 (−11.4 to 16.9)	.71	−2.5 (−19.6 to 14.6)	.78
Affordability of care				
Skipped medication because of cost	8.0 (−3.5 to 19.6)	.18	1.0 (−7.8 to 9.8)	.82
Trouble paying medical bills	−1.9 (−14.3 to 10.4)	.76	−4.2 (−13.8 to 5.5)	.40
Cost-related delay in care	−13.2 (−28.2 to 1.8)	.09	−6.2 (−15.1 to 2.6)	.17

^a^ Results show survey-weighted difference-in-differences estimates for expansion states (Arkansas, Kentucky, and Louisiana) vs Texas. All analyses adjusted for sex, age, race/ethnicity, marital status, education, urban vs rural residence, and state. The sample contained US citizens ages 19 to 64 years with family incomes at or below 138% of the federal poverty level (minus item nonresponse for each specific outcome), except where otherwise noted.

^b^ For the regular care for chronic condition measure, the sample was limited to patients who reported at least 1 of the following conditions: hypertension, heart attack/coronary artery disease, stroke, asthma/chronic obstructive pulmonary disease, diabetes, depression or anxiety, cancer, and substance use disorder.

Table 4 presents rates of financial and nonfinancial barriers to care in 2020 and use of telehealth for the full sample across all 4 states, stratified by race and ethnicity. Overall, 31.2% (95% CI, 25.1%-35.1%) of the sample across the 4 states reported in 2020 that they had delayed care in the past year because of cost, and 15.1% (95% CI, 12.5%-17.7%) had done so for reasons other than cost (respondents were allowed to select 1 or more options, so these groups were not mutually exclusive). The most common reasons for delaying care other than cost were fear of contracting COVID-19 (8.1%), too busy with work or childcare and family (5.2%), and the physician’s office was closed (4.2%). Rates of cost-related delays in care were higher among White individuals, while delays in care because of not wanting to use public transportation were higher among Black and Latinx individuals. Access to telehealth use was not significantly different for White (32.4%) vs Black individuals (26.3%).

**Table 4.** Rates of Financial and Nonfinancial Barriers to Care in 2020

Outcome	Full sample (n = 1804)^a^	Comparison by race and ethnicity			
		Black and Latinx (n = 914)	White (n = 746)	P value	
				Unadjusted difference	Adjusted difference^b^
Delay in care because of cost, % (95% CI)	31.2 (27.6-34.8)	30.1 (25.1-35.1)	33.2 (27.6-38.8)	.42	.03
Delay in care for reasons other than cost, % (95% CI)^c^	15.1 (12.5-17.7)	12.8 (9.5-16.0)	17.9 (13.5-22.2)	.06	.33
Fear of contracting COVID-19, % (95% CI)	8.1 (6.2-10.0)	7.2 (4.8-9.5)	9.0 (5.9-12.2)	.35	.67
Physician’s office closed, % (95% CI)	4.2 (2.8-5.6)	3.1 (1.6-4.5)	5.6 (2.9-8.2)	.08	.45
No access to telehealth, % (95% CI)	3.1 (1.9-4.3)	2.9 (1.4-4.4)	3.0 (1.0-5.0)	.95	.55
Did not want to use public transportation, % (95% CI)	2.8 (1.8-3.9)	3.8 (1.8-5.7)	1.7 (0.8-2.6)	.03	.01
Too busy with work/family, % (95% CI)	5.2 (3.5-6.8)	5.4 (3.1-7.7)	5.3 (2.6-8.0)	.95	.73
Used telehealth in 2020, % (95% CI)	29.0 (25.6-32.3)	26.3 (21.7-30.9)	32.4 (27.2-37.6)	.09	.39

^a^ Full sample includes 143 respondents who were not White, Black, or Latinx. Given sample size limitations, we do not present race-stratified results for those observations, but we did include them in the full sample estimates.

^b^ Adjusted *P* values from survey-weighted logistic regression models adjusting for sex, age, race and ethnicity, marital status, education, urban vs rural residence, presence of a chronic condition (hypertension, heart attack/coronary artery disease, stroke, asthma/chronic obstructive pulmonary disease, diabetes, depression or anxiety, cancer, or substance use disorder), state, and cumulative county-level COVID-19 rates during 2020.

^c^ Respondents were allowed to specify 1 or more reasons for not obtaining care in 2020.

Results were similar when excluding observations from adults who experienced work requirements (Arkansas adults ages 18 to 64 years from 2018 to 2020; data not shown). Pretrend tests of changes from 2018 to 2019, compared between the 3 expansion states vs Texas (nonexpansion state), indicated that 10 of the 12 outcomes did not different significantly by expansion status before the pandemic, while employer-sponsored insurance rates and having a personal physician did (eTable 4 in the Supplement).

## Discussion

In this survey of low-income adults in 4 Southern states, we found worsening access to care in 2020 in all 4 states compared with the 2 years before the COVID-19 pandemic and recession. Coverage rates dropped particularly in Texas, which did not expand Medicaid. Black and Latinx individuals living in Medicaid expansion states were largely insulated from the rise in the uninsured rate compared with those in Texas; however, we did not find this result among White individuals. Previous research indicates that, among eligible adults, enrollment rates in Medicaid are higher among Black and Latinx than White individuals,^17^ which may help explain these findings. Opposition to ACA and Medicaid expansion, which has consistently been higher among White individuals, may also have contributed to this pattern.^18,19^ It is also possible that the 3 states that expanded Medicaid offered more resources and assistance to help indigent populations retain or gain other types of health insurance plans compared with Texas.

While Medicaid expansion was associated with some protective effects on coverage, it was not associated with any differential change in access to care, either in the full sample or in the racial and ethnic subgroup analyses. Both in terms of having a personal physician and regular care for chronic conditions, low-income adults in 2020 experienced significantly worse access to care than during the prepandemic years regardless of state expansion status. While numerous studies show that Medicaid expansion under ACA effectively improved access to care among low-income adults,^7,10,20^ our findings suggest that insurance coverage was not the only factor in the reduced access to care that was reported by respondents in 2020.

The descriptive data on barriers to care support this interpretation. One in 6 adults in our sample (15%) reported delaying medical care during the past year for nonfinancial reasons. This number is similar to other figures that have been reported for different populations, such as 21% among Medicare beneficiaries nationally^21^ and 20% among a national telephone survey.^22^ Fear of contracting COVID-19 was the most commonly reported reason, followed by being too busy with family care/work or the physician’s office being closed. Numerous physician practices closed during the spring of 2020 and the first COVID-19 surge, and others have closed indefinitely because of financial distress.^23^ Meanwhile, transportation barriers were more common among Black and Latinx individuals, who are more likely to be regular users of public transit.

While telehealth has increased rapidly during the pandemic, we found relatively low rates of use overall in the low-income sample (roughly 30% for the year), with a trend to suggest that rates may be lower among Black and Latinx individuals, although it did not reach statistical significance.^24^ Structural barriers, such as lack of broadband internet, lack of usable devices, and language barriers, have been identified as contributors to this disparity, and addressing these sources of inequity should be a focus for policy makers.^25^ Furthermore, there are concerns that safety net clinicians who are more likely to care for low-income patients are less likely to offer telehealth services.^26^ We did not find any significant differences in telehealth use across the 4 states; state coverage policies for telehealth in Medicaid in these 4 states were generally similar in 2020.^27^

### Limitations

Our analysis has limitations. First, the data set comes from a telephone survey with a response rate that was lower than that of most government surveys, although similar to that of the US Census Bureau’s new Pulse Survey that was designed to assess responses to the pandemic (which had weekly response rates ranging from 1.3% to 10.3% in 2020)^28^ and other telephone and internet-based surveys.^29^ In addition, there may be a bias in the type of person who is more likely to respond to the survey compared with those who did not respond. We used survey weights to mitigate nonresponse bias, and prior research using our survey methods has produced state estimates of health insurance coverage and other outcomes similar to those from the American Community Survey.^12^ Nonetheless, it is possible that nonresponse bias, as well as recall bias, which both may be exacerbated by the circumstances of the COVID-19 pandemic, may have affected the results, although the overall response rate in 2020 was similar to the 2019 response rate.

Second, the study focused on 4 states. All 4 states are in the South and have relatively high poverty rates and cumulative COVID-19 infection rates in the middle to upper tier of the US (ranking 14th, 23rd, 29th, and 30th based on total 2020 cases). It is unclear if our results generalize to states in other parts of the country with lower poverty rates and markedly different COVID-19 infection rates. Third, when determining the health insurance plans, some respondents limited their response as being in an other health insurance plan despite prompting from interviewers for a more specific categorization.

Third, our sample was limited to US citizens. Immigrant communities have been adversely affected by the COVID-19 pandemic,^3^ and policy changes under the Trump administration, including a broader definition of the public charge law that uses participation in public programs as a factor in permanent residency status decisions, have been associated with worsening coverage and lack of participation in Medicaid.^30,31^ Future research with alternative data sources will be needed to assess the coverage effects of the pandemic among noncitizens.

Finally, the difference-in-difference analysis of coverage and access to care identifies associations between the Medicaid expansion and changes in these outcomes during the COVID-19 pandemic, but we cannot rule out other time-varying confounders associated with the pandemic response that may affect these results. In particular, if states that expanded Medicaid implemented other policies associated with COVID-19 and/or the economic recession, these changes could confound our findings and attribution of differences in coverage and access to Medicaid. However, we are unaware of any major 2020 policy initiatives in these states that differed along these lines. Finally, the descriptive analysis of barriers to care was cross-sectional in nature; therefore we cannot conclude a causal relationship between the patterns of barriers and racial groups..

## Conclusions

During the COVID-19 pandemic and 2020 recession, uninsured rates rose among low-income adults, but in states with Medicaid expansion, that association was diminished among Black and Latinx individuals. Nonetheless, access to care across multiple measures worsened across all groups in 2020 regardless of Medicaid expansion status, which appears to reflect new nonfinancial barriers to care that were created by the pandemic.

## Notes

Supplement. **eMethods 1. **Additional Details on Survey Approach **eMethods 2. **Survey Questions **eMethods 3. **Regression Equations **eTable 1: **Unadjusted Outcome Measures, By Year and Expansion Status **eTable 2: **Unadjusted Outcome Measures Among Black and Latino Respondents, By Year and Expansion Status **eTable 3: **Unadjusted Outcome Measures Among White Respondents, By Year and Expansion Status **eTable 4: **Pre-Trend Testing for 2019 vs. 2018 in Difference-in-Differences Model
