# Supplementary material for: COVID-19–Related Insurance Coverage Changes and Disparities in Access to Care Among Low-Income US Adults in 4 Southern States
Source: JAMA Health Forum. 2021 Aug 13;2(8):e212007. doi: 10.1001/jamahealthforum.2021.2007 (PMC8796874; doi:10.1001/jamahealthforum.2021.2007)
Supplement: Supplement. — eMethods 1. Additional Details on Survey Approach eMethods 2. Survey Questions eMethods 3. Regression Equations eTable 1: Unadjusted Outcome Measures, By Year and Expansion Status eTable 2: Unadjusted Outcome Measures Among Black and Latino Respondents, By Year and Expansion Status eTable 3: Unadjusted Outcome Measures Among White Respondents, By Year and Expansion Status eTable 4: Pre-Trend Testing for 2019 vs. 2018 in Difference-in-Differences Model [file jamahealthforum-e212007-s001.pdf]

Supplemental Online Content

Figueroa JF, Khorrami P, Bhanja A, Orav EJ, Epstein AM, Sommers BD. COVID-19–related insurance coverage changes and disparities in access to care among low-income US adults in 4 Southern states. *JAMA Health Forum*. 2021;2(8):e212007. doi:10.1001/jamahealthforum.2021.2007

eMethods 1. Additional Details on Survey Approach
eMethods 2. Survey Questions
eMethods 3. Regression Equations
eTable 1: Unadjusted Outcome Measures, By Year and Expansion Status
eTable 2: Unadjusted Outcome Measures Among Black and Latino Respondents, By Year and Expansion Status
eTable 3: Unadjusted Outcome Measures Among White Respondents, By Year and Expansion Status
eTable 4: Pre-Trend Testing for 2019 vs. 2018 in Difference-in-Differences Model

This supplemental material has been provided by the authors to give readers additional information about their work.

© 2021 Figueroa JF et al. *JAMA Health Forum.*

eMethods 1. Additional Details on Survey Approach

We employed a non-partisan survey vendor, SSRS, to conduct a random-digit dialing (RDD) survey. Inclusion criteria and sample sizes are noted in the main paper Methods section. While most respondents were newly-identified individuals contacted via random-digit dialing who screened positive for the study's inclusion criteria, to reach respondents more efficiently, a small portion of respondents were reached by calling back respondents who completed an interview on a different survey in recent years – the SSRS Omnibus poll, a national weekly dual-frame (cell phone and landline) RDD telephone poll.

All interviews were conducted using the CATI system. The CATI system ensured that questions followed logical skip patterns and that complete dispositions of all call attempts were recorded. CATI interviewers received both formal training and written materials on the survey. The written materials were provided prior to the beginning of the field period and included an annotated questionnaire that contained information about the goals of the study as well as the pronunciation of key terms. Additional written materials detailed potential obstacles to be overcome in order to get good answers to questions, respondent problems that could be anticipated ahead of time, as well as strategies for addressing them.

Interviewer training was conducted both prior to the study pretest and immediately before the survey was officially launched. Call center supervisors and interviewers were walked through each question in the questionnaire. Interviewers were given instructions to help them maximize response rates and ensure accurate data collection. Interviewers were also monitored throughout the study and project staff provided feedback to interviewers throughout the survey period.

In order to maximize survey response, SSRS enacted the following procedures during the field period:

- Each non-responsive number not already set up with a callback (i.e. answering machines, no answers and busy) was contacted up to 4 times, varying the times of day, and the days of the week that callbacks were placed using a programmed differential call rule.

© 2021 Figueroa JF et al. *JAMA Health Forum.*

- Interviewers explained the purpose of the study, its relevance to respondents, and clarified that this was not a sales call.
- All numbers with initial refusals were re-contacted by interviewers who specialize in refusal conversion.
- Cell phone respondents, who mentioned costs of airtime, were offered $10 to reimburse them for these costs. Callback respondents were all offered $10 for participating in the survey.

Response rates were calculated based on the *American Association for Public Opinion Research's* RR3 definition. Below are the response rates by year, state, and phone type. The overall response rate for the full study period was 11%.

State and Phone Type	**2018**	**2019**	**2020**
Arkansas Cell Phone	17%	7%	6%
Arkansas Land Line	11%	9%	12%
Arkansas Callbacks	28%	31%	19%
Arkansas TOTAL	15%	8%	7%
Kentucky Cell Phone	10%	9%	7%
Kentucky Land Line	19%	14%	23%
Kentucky Callbacks	26%	15%	*NA*
Kentucky TOTAL	12%	11%	10%
Texas Cell Phone	10%	5%	6%
Texas Land Line	19%	3%	29%
Texas Callbacks	20%	16%	21%
Texas TOTAL	13%	7%	10%
Louisiana Cell Phone	16%	9%	7%
Louisiana Land Line	15%	20%	26%
Louisiana Callbacks	23%	17%	34%
Louisiana TOTAL	16%	12%	9%
TOTAL	14%	8%	9%

We re-weighted the dataset to ensure an accurate representation of the target population in each state. The survey weights accounted for differential response rates across demographic groups, cellphone vs. landline use, and the probability of selection within households, using population benchmarks from the American Community Survey (ACS) for age, gender, race/ethnicity, marital status, geographic region within-state (by region), population density; benchmarks for cellphone vs. landline status came from the National Health Interview Survey.

© 2021 Figueroa JF et al. *JAMA Health Forum.*

Insurance type was categorized into mutually-exclusive categories using the following hierarchy: Medicaid, Medicare, Marketplace, Employer-Sponsored Insurance (ESI), and other insurance.

eMethods 2. Survey Questions

The following survey items were used for our study outcomes:

Health Insurance

Ask all

		Marketplace Name	Medicaid Program
AR	Arkansas	The Arkansas Health Connector, also called "My Arkansas Insurance" OR The HealthCare.gov website	Medicaid, the Private Option, or the Arkansas Works health insurance program
KY	Kentucky	KYnect (pronounced: Connect) OR The HealthCare.gov website	Medicaid, Kentucky Partnership Program, or Kentucky HEALTH
TX	Texas	The HealthCare.gov website	Medicaid, STAR, or STAR+PLUS
LA	Louisiana	The HealthCare.gov website	Medicaid, Healthy Louisiana, or Bayou Health

1) I am going to read a few common types of health insurance. For each one, please tell me 'yes' if you currently have it and 'no' if you don't. You can answer 'yes' more than once.

a. [State Medicaid Plan Name] (INTERVIEWER NOTE: Clarify, if needed, "Medical Assistance or government-assistance plan for those with low incomes or a disability")
b. Medicare (INTERVIEWER NOTE: Clarify, if needed, "for people 65 and older, or people with certain disabilities")
c. A military health care plan, such as TRI-CARE, CHAMPUS, or CHAMP-VA
d. A health plan you got through an employer or union (INTERVIEWER NOTE: This also includes through a spouse's employer or union)
e. A health insurance plan that you signed up for through [State Marketplace Name] or a health insurance Marketplace created by the national health reform law. (INTERVIEWER NOTE: If respondent says "do you mean Obamacare or ACA," then say: "The national health reform law is sometimes referred to as Obamacare or the Affordable Care Act")
f. A health plan that you bought directly from an insurance company, not through an employer or union, and not through a health insurance Marketplace
g. Some other kind of health insurance I haven't already mentioned (SPECIFY TYPE:____)

If no to all:

h. Does this mean you have no health insurance of any kind?

If respondent does not currently have Medicaid:

i. In the past 12 months, did you ever lose Medicaid coverage or drop out of the program?

Health Care Access and Utilization

2) Is there one person you think of as your personal doctor or health care provider, or not?

© 2021 Figueroa JF et al. *JAMA Health Forum.*

3) What is the **main** location you normally go to, to receive medical care?

4) At any time in the last year, have you waited to seek medical care or chosen not to seek medical care for an illness, injury, or condition because you couldn't afford it?

5) At any time in the last year, have you waited to seek medical care or chosen not to seek medical care for an illness, injury, or condition for a reason <u>*besides*</u> that you couldn't afford it?

If yes:

a. Did you wait to seek medical care or choose not to seek medical care because you were afraid of contracting coronavirus?
b. Did you wait to seek medical care or choose not to seek medical care because your doctor's office was closed?
c. Did you wait to seek medical care or choose not to seek medical care because you didn't have access to telehealth or telemedicine?
d. Did you wait to seek medical care or choose not to seek medical care because you couldn't or didn't want to use public transportation to get an appointment? (INTERVIEWER NOTE: This includes public transportation, ride-share, or a cab)
e. Did you wait to seek medical care or choose not to seek medical care because you were too busy with work, childcare, or caring for a family member?

6) At any time in the last year, have you ended up taking less medication than was prescribed for you because you couldn't afford it? This includes skipping doses, or taking a smaller dose than was prescribed, or not filling a prescription right away.

7) In the past year, have you either had to borrow money, or skip paying medical bills, or skip paying other bills as a result of medical costs?

8) In the past year, have you used telehealth, video, or phone services to receive medical care instead of visiting in person?

<u>Health Status</u>

9) I am going to read a list of medical conditions. For each, please indicate if you have ever been told by a doctor or other health professional that you have had that condition.

a. High blood pressure
b. A heart attack, coronary artery disease, or heart failure
c. Asthma, chronic bronchitis, COPD, or emphysema
d. Diabetes
e. Depression or anxiety
f. Cancer, except for skin cancer
g. Alcoholism or drug addiction

If yes to any:

h. You mentioned that you have (a/more than one) health problem or diagnosed condition. In the last 12 months, have you regularly seen or communicated with a doctor or other healthcare provider in order to treat (your condition/any of these conditions)? This does not include the Emergency Room.

eMethods 2. Regression Equations

1) Difference-in-Difference (DD) Model of Coverage and Access to Care – Tables 2 and 3:

$$Uninsured_{icst} = \beta_0 + \beta_1 X_i + \beta_2 State_s + \beta_3 Year2020_t + \beta_4 ExpansionState_s * Year2020_t + \varepsilon_{icst}$$

Eqn. 1

where i indexed individuals, c county, s state, and t year. X_i was a vector of demographics (gender, race/ethnicity, marital status, education, urban vs. rural status, and age group). β_2 and β_3 capture the direct effects of State (with fixed effects for each) and the post-pandemic period. β_4 is the DD estimate for the change in outcome in 2020 (compared to 2018-2019) associated with Medicaid expansion, comparing to the control states. This was a linear regression model using robust standard errors clustered at the county level.

2) Pre-COVID Testing of Parallel Trends Between Expansion and Non-Expansion States from 2018 to 2019– Appendix Table 4:

This model assessed whether trends in coverage and access to care were similar for the expansion and non-expansion states prior to the 2020 pandemic. Using the 2018-2019 data only, we replicated Equation 1, but with 2019 as a "placebo" year:

$$Uninsured_{icst} = \beta_0 + \beta_1 X_i + \beta_2 State_s + \beta_3 Year2019_t + \beta_4 ExpansionState_s * Year2019_t + \varepsilon_{icst}$$

Eqn. 2

3) Demographic Predictors of Barriers to Care and Telehealth Use – Table 4:

This model used cross-sectional logistic regression models to identify factors associated with barriers to care and telehealth use in the 2020 survey:

© 2021 Figueroa JF et al. *JAMA Health Forum.*

$$Telehealth_{ics} = \beta_0 + \beta_1 Age_i + \beta_2 Education_i + \beta_3 Marital\ Status_i + \beta_4 Rural_i + \beta_5 Race/Ethnicity_i + \beta_6 ChronicCondition + \beta_7 Sex + \beta_8 CountyCOVIDRate_c + \beta_9 State_s * Year2019_t + \varepsilon_{ics}$$

Eqn. 3

© 2021 Figueroa JF et al. *JAMA Health Forum.*

eTable 1: Unadjusted Outcome Measures, By Year and Expansion Status

OUTCOME	AR, KY, and LA (Medicaid expansion states)		Texas (non-expansion state)	
	2018-2019	2020	2018-2019	2020
Coverage				
Uninsured	11.4% (9.5, 13.2)	10.8% (8.0, 13.6)	30.5% (26.3, 34.7)	34.5% (29.8, 39.2)
Medicaid or Marketplace	59.7% (57.0, 62.4)	62.6% (58.2, 67.1)	35.8% (31.5, 40.1)	34.4% (29.7, 39.0)
Employer Sponsored Insurance	13.3% (11.4, 15.3)	9.7% (7.0, 12.4)	19.7% (16.1, 23.4)	18.1% (14.0, 22.3)
Other Health Insurance	13.5% (11.8, 15.2)	13.6% (10.6, 16.5)	11.3% (8.5, 14.1)	10.2% (7.5, 13.0)
Access to Care				
Has a personal doctor	63.9% (61.1, 66.6)	59.7% (55.1, 64.3)	52.3% (47.7, 56.8)	45.4% (40.4, 50.3)
Usual source of care	85.6% (83.6, 87.6)	82.6% (79.0, 86.3)	75.3% (71.3, 79.4)	70.7% (66.1, 75.3)
Regular care for chronic condition[a]	72.8% (69.9, 75.8)	69.4% (64.5, 74.3)	61.4% (55.9, 67.0)	60.1% (53.9, 66.3)
Affordability of Care				
Skipped medication due to cost	29.5% (26.9, 32.0)	21.2% (17.7, 24.6)	34.3% (30.0, 38.7)	22.8% (18.6, 27.1)
Trouble paying medical bills	32.1% (29.5, 34.7)	22.5% (19.0, 25.9)	36.3% (31.9, 40.8)	30.7% (26.2, 35.2)
Cost-related delay in care	29.5% (26.9, 32.1)	20.0% (16.6, 23.3)	37.7% (33.3, 42.1)	35.9% (31.1, 40.7)

Notes: The sample contained 7515 U.S. citizens ages 19-64, with family incomes at or below 138% of the federal poverty level (minus item non-response for each specific outcome), except where otherwise noted below. Results reflect survey weighting.

[a]For the "regular care for chronic condition" measure, the sample was limited to patients reporting at least one of the following conditions: hypertension, heart attack/coronary artery disease, stroke, asthma/COPD, diabetes, depression or anxiety, cancer, and substance use disorder.

© 2021 Figueroa JF et al. *JAMA Health Forum.*

eTable 2: Unadjusted Outcome Measures Among Black And Latino Respondents, By Year and Expansion Status

OUTCOME	AR, KY, and LA (Medicaid expansion states)		Texas (non-expansion state)	
	2018-2019	2020	2018-2019	2020
Coverage				
Uninsured	9.8% (6.8, 12.8)	9.3% (5.9, 12.6)	29.0% (23.6, 34.4)	37.5% (31.6, 43.4)
Medicaid or Marketplace	67.8% (63.2, 72.3)	66.6% (60.0, 73.3)	38.4% (32.7, 44.1)	37.6% (31.5, 43.7)
Employer Sponsored Insurance	12.0% (8.8, 15.3)	11.7% (6.4, 17.0)	18.5% (14.0, 23.1)	16.5% (11.6, 21.4)
Other Health Insurance	9.9% (7.5, 12.2)	11.7% (7.3, 16.1)	10.9% (7.2, 14.6)	6.9% (4.4, 9.3)
Access to Care				
Has a personal doctor	59.4% (54.3, 64.6)	55.9% (48.9, 62.9)	49.3% (43.5, 55.2)	43.3% (37.2, 49.5)
Usual source of care	85.2% (81.6, 88.9)	82.3% (76.9, 87.8)	74.9% (69.6, 80.2)	71.3% (65.6, 77.0)
Regular care for chronic condition	74.6% (69.6, 79.7)	70.2% (63.6, 76.8)	60.4% (53.0, 67.8)	58.9% (50.6, 67.2)
Affordability of Care				
Skipped medication due to cost	23.6% (19.2, 28.0)	17.1% (12.3, 21.9)	30.4% (24.9, 35.9)	21.9% (16.6, 27.2)
Trouble paying medical bills	28.4% (23.8, 33.0)	19.7% (15.0, 24.4)	38.3% (32.4, 44.2)	32.9% (27.1, 38.8)
Cost-related delay in care	23.2% (18.6, 27.7)	15.3% (10.8, 19.8)	36.5% (30.8, 42.3)	33.6% (27.6, 39.6)

Notes: The sample contained 3,037 U.S. citizens ages 19-64, with family incomes at or below 138% of the federal poverty level (minus item non-response for each specific outcome), who reported their race as Black and/or ethnicity as Latino or Hispanic. Results reflect survey weighting.

© 2021 Figueroa JF et al. *JAMA Health Forum.*

eTable 3: Unadjusted Outcome Measures Among White Respondents, By Year and Expansion Status

OUTCOME	AR, KY, and LA (Medicaid expansion states)		Texas (non-expansion state)	
	**2018-2019**	**2020**	**2018-2019**	**2020**
Coverage				
Uninsured	11.7% (9.3, 14.1)	10.5% (6.8, 14.1)	31.9% (24.8, 39.1)	29.1% (20.8, 37.3)
Medicaid or Marketplace	56.4% (52.9, 59.9)	61.4% (55.4, 67.3)	33.9% (26.6, 41.2)	30.7% (22.9, 38.5)
Employer Sponsored Insurance	14.0% (11.4, 16.5)	8.7% (5.6, 11.9)	21.5% (14.9, 28.0)	21.5% (13.6, 29.3)
Other Health Insurance	15.1% (12.8, 17.4)	14.7% (10.6, 18.8)	11.7% (7.0, 16.4)	14.9% (8.9, 20.8)
Access to Care				
Has a personal doctor	66.7% (63.3, 70.1)	61.6% (55.4, 67.8)	57.3% (49.6, 64.9)	50.6% (41.7, 59.5)
Usual source of care	86.2% (83.7, 88.8)	83.7% (79.0, 88.5)	73.7% (66.6, 80.7)	72.3% (64.2, 80.3)
Regular care for chronic condition	72.1% (68.4, 75.9)	70.0% (63.4, 76.6)	65.3% (56.4, 74.2)	61.9% (51.8, 72.1)
Affordability of Care				
Skipped medication due to cost	32.1% (28.8, 35.3)	21.9% (17.1, 26.7)	42.1% (34.5, 49.8)	25.5% (17.8, 33.3)
Trouble paying medical bills	33.5% (30.2, 36.7)	23.4% (18.6, 28.3)	34.7% (27.3, 42.0)	28.4% (20.7, 36.0)
Cost-related delay in care	32.3% (29.0, 35.6)	21.8% (17.0, 26.5)	38.0% (30.6, 45.4)	41.4% (32.6, 50.2)

Notes: The sample contained 3,889 U.S. citizens ages 19-64, with family incomes at or below 138% of the federal poverty level (minus item non-response for each specific outcome), who reported their race and ethnicity as White non-Latino. Results reflect survey weighting.

© 2021 Figueroa JF et al. *JAMA Health Forum.*

eTable 4: Pre-Trend Testing for 2019 vs. 2018 in Difference-in-Differences Model

OUTCOME	AR, KY, and LA (Medicaid expansion states)		Texas (non-expansion state)		Difference-in-Differences Estimate[a]		
	2019 vs. 2018	p-value	2019 vs. 2018	p-value	Net Change	95% CI	p
Coverage							
Uninsured	0.3%	0.895	-2.0%	0.631	2.2%	-6.7, 11.1	0.625
Medicaid or Marketplace	0.4%	0.856	1.3%	0.759	-0.8%	-10.0, 8.5	0.863
Employer Sponsored Insurance	-2.9%	0.123	4.5%	0.116	-7.5%	-14.1, -0.7	0.030
Other Health Insurance	2.0%	0.256	-0.7%	0.810	2.7%	-3.7, 0.91	0.408
Access to Care							
Has a personal doctor	0.9%	0.756	13.4%	0.007	-12.5%	-23.6, -1.4	0.028
Usual source of care	0.5%	0.804	3.7%	0.299	-3.2%	-11.1, 4.7	0.425
Regular care for chronic condition[b]	1.3%	0.658	10.2%	0.12	-8.9%	-18.7, 0.8	0.73
Affordability of Care							
Skipped medication due to cost	2.6%	0.274	-1.7%	0.714	4.2%	-5.8, 14.3	0.410
Trouble paying medical bills	-3.1%	0.239	-2.6%	0.523	-0.5%	-9.9, 8.9	0.921
Cost-related delay in care	2.0%	0.429	-2.1%	0.630	4.1%	-5.6, 13.7	0.410

a – Results show survey-weighted difference-in-differences estimates for expansion states (Arkansas, Kentucky, and Louisiana) versus Texas, for 2019 vs. 2018. All analyses adjusted for sex, age, race/ethnicity, marital status, education, urban vs. rural residence, and state.

b – Sample limited to patients reporting at least one of the following conditions: hypertension, heart attack/coronary artery disease, stroke, asthma/COPD, diabetes, depression or anxiety, cancer, and substance use disorder).

© 2021 Figueroa JF et al. *JAMA Health Forum.*
